# A dataset of functional traits for compound pinnate leaves of plants in the Huangshui River Valley of Qinghai Province, China

**DOI:** 10.3897/BDJ.11.e106254

**Published:** 2023-07-26

**Authors:** Qian Wang, Anselmo Nogueira, Ji-Zhong Wan, Chun-Jing Wang, Lan-ping Li

**Affiliations:** 1 College of Agriculture and Animal Husbandry, Qinghai University, Xining City, Qinghai Province, China College of Agriculture and Animal Husbandry, Qinghai University Xining City, Qinghai Province China; 2 Centro de Ciências Naturais e Humanas, Universidade Federal do ABC, São Bernardo do Campo – SP, Brazil Centro de Ciências Naturais e Humanas, Universidade Federal do ABC São Bernardo do Campo – SP Brazil; 3 State Key Laboratory of Plateau Ecology and Agriculture, Qinghai University, Xining City, Qinghai Province, China State Key Laboratory of Plateau Ecology and Agriculture, Qinghai University Xining City, Qinghai Province China

**Keywords:** compound pinnate leaves, dataset, functional traits, Huangshui River basin, Qinghai Province

## Abstract

**Background:**

Here, we present data collected from the Qinghai–Tibet Plateau that describes the variation of leaf functional traits across 32 plant species and could be used to investigate plant community functioning and predict the impact of climate change on biogeochemical cycles. The sampling area is located in Huangshui River Valley, in the southeast of Qinghai Province, China (36° 19′ to 36° 53′ N, 100° 59′ to 102° 48′ E). The area contains an alpine meadow typical of the Qinghai–Tibet Plateau.

**New information:**

This dataset includes field survey data on the functional properties of compound leaves from herbaceous species in the Huangshui River Basin of Qinghai Province, China, at altitudes from 1800 m to 4000 m in the summer of 2021. Data were collected from 326 plots, including 646 data points of compound leaf plants, spanning 32 compound leaf plant species belonging to 14 genera and four families. The study species were chosen from 47 families, 165 genera and 336 species present in the plots and all compound leaf plants were chosen within each plot. We picked the parts containing leaves, petioles and rachis from the study plants and separated the leaves from the plants. The cut compound leaf part was a leaflet, while the petiole and rachis were linear elements. The dataset includes information about the leaflet trait variation (i.e. leaflet area, leaflet dry mass, specific leaflet area and leaflet nitrogen content per unit dry mass) and linear elements' biomass and nitrogen content per unit dry mass (i.e. both petiole and rachis) of 646 compound leaves. This dataset can be used to analyse the evolution of leaf traits and the basic functioning of ecosystems. Moreover, the dataset provides an important basis for studying the species distribution and protection of biodiversity of the Qinghai–Tibet Plateau and evaluating ecosystem services. These data also support the high-quality development of the Yellow River Basin and have empirical and practical value for alpine biodiversity protection and ecosystem management.

## Introduction

Plant organisms are associated with the environment by quantifying the functional characteristics of plants (Lavorel and Garnier 2002). Plant functional traits can be used as predictors of ecosystem services (Garnier and Navas 2012) and are often used to assess plant adaptability to the environment (He et al. 2020). Leaf functional traits are crucial in ecosystem services (Kissling et al. 2019) and are directly linked to the performance of photosynthesis and respiration (Xiong and Jiao 2019). The compound leaves of plants result from the subdivision of simple leaves into individual leaflets (Sinha 1997, Kim et al. 2003). Each leaflet of a compound leaf is the main photosynthetic organ of compound leaf plants (Oliveira et al. 2017). The blade surface area can affect the water loss caused by wind resistance and evaporation. The smaller the surface area, the lower the water loss caused by wind resistance and evaporation (Anten et al. 2010). The leaflets of compound pinnate leaves can regulate resource and energy flux (Hadley and Smith 1990, Brus et al. 2011). Therefore, leaflet trait variation is crucial to ecosystem functioning and services (Hulshof and Swenson 2010, Blue et al. 2015). A better understanding of the relationship between plant traits and trends in trait variation across species is conducive to better management of ecosystems (Wright et al. 2004, Donovan et al. 2011, Osnas et al. 2013, Liu et al. 2020).

The global climate change is the most serious challenge facing mankind at present, promoting the loss of biodiversity in an unprecedented way on Earth. Owing to its unique altitude and climate conditions, the ecosystem of the Qinghai–Tibet Plateau is very sensitive to global climate change and is one of the most sensitive regions around the world (Hu et al. 2009). Ecological protection and high-quality development of the Yellow River Basin is a major matter of national strategic importance in China. Huangshui River is the largest tributary of the upper reaches of the Yellow River, located in the east of Qinghai Province, China. The Huangshui River Basin is an ecologically fragile area of the Qinghai–Tibet Plateau and its ecosystem functions and services are greatly threatened. The protection of plant diversity in the Huangshui River Basin should not only meet the needs of biodiversity protection in the Qinghai–Tibet Plateau, but also support the maintenance and high-quality development of the ecosystems of the Yellow River Basin.

We hope that this large dataset of plant compound leaf functional traits from the Huangshui River Valley in Qinghai Province provides a starting basis for studying the species distribution, evaluating the area’s ecosystem functions and services and protecting the alpine biodiversity of the Qinghai–Tibet Plateau.

## Sampling methods

### Study extent

**Figure 1.**
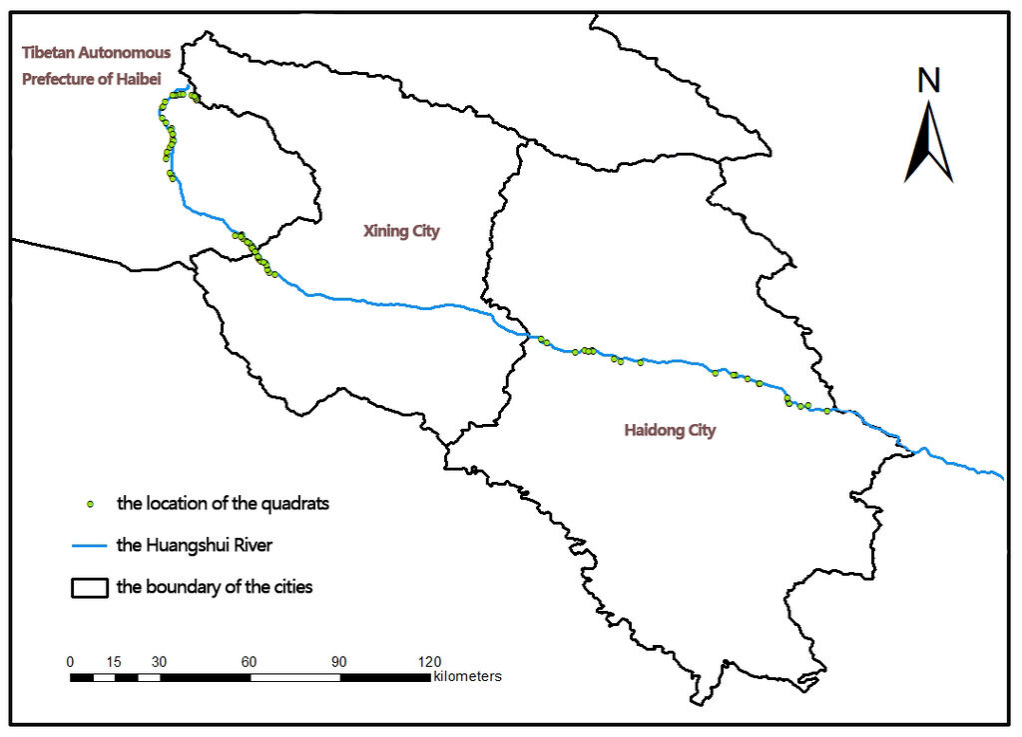
Distribution map of plots.

The study was conducted in the Huangshui River Valley, which is in the east of Qinghai Province, China, on the eastern end of the Qinghai–Tibet Plateau. Based on the scheme of Fang et al. (2009), 326 sampling plots of 1 m^2^ of area were established in the Huangshui River watershed at altitudes from 1,800 m to 4,000 m (Fig. 1). The field survey was conducted from June 2021 to September 2021. From 326 study plots, we collected all the leaves from at least five individuals for each plant species.

### Sampling description

The 326 plots (1 m × 1 m) were distributed systematically in the landscape (Fig. 1). The slope within plots was stable, so the influence of microenvironment heterogeneity on the variation of functional traits was successfully limited. In each plot, we sampled all herbaceous plants bearing compound leaves. A total of 646 occurrences were recorded (mean _(plants/plots)_ = 1.98 ± 1.16), including 32 plant species, 14 genera and four families. The leaf samples were stored in a cool box in the dark until further processing a total of 646 in the laboratory. The time from sample collection to the laboratory procedures was less than 18 h. The dataset records Plot No., Family name, Genus name, Species name, Authors’ name, Classification System, Habitat, Life cycle or leaf phenology type, Coverage, Leaflet area (LA), Specific leaflet area (SLA), Leaflet dry mass (LM), Leaflet nitrogen content per unit dry mass (LN), Petiole and rachis dry mass and Petiole and rachis nitrogen content per unit dry mass. We selected LA (cm^2^), LM (g), SLA (cm^2^/g), and LN (mg/g) as related leaflet functional traits, because they are generally considered to be related to major ecological strategy axes.

### Quality control

In the data collection stage, we invited botanists for identification training of relevant compound leaf herb species and all data collection personnel started field investigation only after completion of the training. The reference books mainly included Flora Republicae Popularis Sinicae (Editorial Committee of the Flora of China, Chinese Academy of Sciences 1999), Flora Qinghaiica (ECFQ 1996, Editorial Committee of the Flora Qinghaiica 1996), Atlas of Vascular Plants in Hainan Tibetan Autonomous Prefecture (Zhou et al. 2020), Illustration of Grassland Plants in Gansu Province (Zhao 2019), Wild Flowers of Qinghai-Tibet Plateau (Niu et al. 2018) and Atlas of Common Plants in Alpine Sandy Land (Jia and Zhu 2017). In the data processing stage, the identification of all herbaceous plants strictly referred to the classification characteristics described in various books and related publications and all plant species in question were confirmed by experts. With the rapid development of plant systematics, a large number of new species have been described in recent years. The present dataset does not include newly-published species.

Before we quantified the nitrogen content per unit dry mass of leeflet and linear elements (petiole and rachis) samples by Kjeldahl (acid) digestion, the samples of each compound leaf were stored in a clean numbered bag and frozen in -80℃ liquid nitrogen to ensure the dryness of the samples and to reduce experimental error.

### Step description

Following the methods of Cornelissen et al. (2003), Pérez-Harguindeguy et al. (2013)and Pérez-Harguindeguy et al. (2016), we initially collected at least five fresh compound leaf samples from each plant. Each compound leaf was carefully divided into petiole, rachis and leaflet portions. Parts coming from the same leaf were kept named so that we would not mix parts of different leaves or plants. Leaflet area (LA) was calculated in cm^2^ by scanning each fresh leaf digitally and then analysing the images using Easy leaf Area (Easlon and Bloom 2014). After at least 72 h in the drying oven at 65°C, we measured the dry mass of petioles, rachis and leaflets of each leaf in milligrams (mg). SLA was calculated as the ratio of leaf area (cm^2^) to dry leaf mass in grams (g). Finally, we used Kjeldahl (acidic) digestion to quantify the nitrogen content (N) in the leaflets, linear elements (both petiole and rachis), followed by colorimetric (flow-injection) analysis. Subsequently, we obtained the nitrogen content per unit dry mass of leaflet and linear elements (petiole and rachis) by dividing the total nitrogen content (in milligrams, mg) by the summed total dry mass of all leaflets and linear elements per leaf (in grams, g), respectively.

## Geographic coverage

### Description

The Huangshui River Valley in Qinghai Province, China surrounds the Huangshui River, the largest tributary of the upper reaches of the Yellow River in China. It also carries the main run-off of the upper reaches of the Yellow River, maintains the balance of water resources of the Yellow River and plays a role as an ecological protection barrier. Huangshui River is located in the Baohutu Mountains in the east of Qinghai Province, China, serving as the junction of the Qinghai–Tibet Plateau and the Loess Plateau. The total area of the Huangshui River Basin is about 16,100 km^2^. Its wide area and large altitude drop shape its unique hydrological geomorphology and plant community composition.

### Coordinates

 and 36° 19′ to 36° 53′ Latitude; and 100° 59′ to 102° 48′ Longitude.

## Taxonomic coverage

### Description

The general taxonomic coverage includes four families, 14 genera and 32 plant species. Although the species we found were approximately 33.7% of those previously recorded (Huang et al. 2021), we have provided the geographical coordinates of all species that we observed. It was beyond our intended research scope to conduct a complete inventory of compound leaved herbs in the Huangshui River Valley.

### Taxa included

**Table:** 

Rank	Scientific Name	
kingdom	Plantae	
family	Leguminosae	
family	Rosaceae	
family	Ranunculaceae	
family	Lamiaceae	
genus	*Oxytropis*	
genus	*Vicia*	
genus	*Astragalus*	
genus	*Potentilla*	
genus	*Sibbaldianthe*	
genus	*Thalictrum*	
genus	*Coluria*	
genus	*Dasiphora*	
genus	*Medicago*	
genus	*Hedysarum*	
genus	*Sphaerophysa*	
genus	*Tibetia*	
genus	*Salvia*	
genus	*Melilotus*	

## Temporal coverage

### Notes

Data collection dates: 2021.06.27 to 2021.08.20.

## Usage licence

### Usage licence

Creative Commons Public Domain Waiver (CC-Zero)

## Data resources

### Data package title

A dataset of functional traits for compound pinnate leaves of plants in the Huangshui River Valley of Qinghai Province, China.

### Resource link

https://www.scidb.cn/anonymous/YWlFYml5

### Number of data sets

2

### Data set 1.

#### Data set name

Plot information

#### Description

This dataset records Plot No., Administrative Position, Longitude, Latitude, Elevation, Disturbance degree, Vegetation type, Plot coverage, Slope and Time. Each line represents one plot.

**Data set 1.** 

Column label	Column description
Plot No.	We use the combination of the abbreviation of the administrative location and the sampling geographic location number to represent the number of each plot.
Administrative Position	Administrative Position includes county, prefecture-level city (Autonomous Prefecture), province and country. "county": The full, unabbreviated name of the next smaller administrative region than prefecture-level city and Autonomous Prefecture. "prefecture-level city (Autonomous Prefecture)": The name of the prefecture-level city and Autonomous Prefecture of Qinghai Province in which the Location occurs. In our case, it is always Xining City, Haidong City and Tibetan Autonomous Prefecture of Haibei. "province ": The name of the province which the Location occurs. In our case, it is always Qinghai Province. "country ": The name of the country unit in which the Location occurs. In our case, it is always China.
Longitude (°E)	Longitude in decimal degrees, datum WGS84.
Latitude (°N)	Latitude in decimal degrees, datum WGS84.
Elevation (m)	The vertical distance of the ground above sea level. China uses the height from the mean sea level of the Yellow Sea (1985 National Elevation Datum) as the standard for calculation.
Disturbance degree	The degree of interference by human activities. In our study, the traces left by human interference, such as human footprints, garbage residue and combustion residue, are divided into Weak, Medium and Strong according to the degree.
Vegetation type	Vegetation physiognomy characterised by the dominant plants in the plot. In our case, it includes Grassland, Shrub and Forest.
Plot coverage (%)	This refers to the ratio of the projected area of all plants (herbs and woody plants) in the Plot to the total area of land.
Slope (°)	Slope of the land measured with a clinometer in degrees.
Date (yyyy-mm-dd)	Date of data survey.

### Data set 2.

#### Data set name

Plot composition

#### Description

This dataset records Plot No., Plot No.- Species code, Family name, Genus name, Species name, Authors’ name, Classification System, Habitat, Life cycle or leaf phenology type, Coverage, Leaflet area (LA), Specific leaflet area (SLA), Leaflet dry mass (LM), Leaflet nitrogen content per unit dry mass (LN), Petiole and rachis dry mass and Petiole and rachis nitrogen content per unit dry mass. Each line represents one leaf.

**Data set 2.** 

Column label	Column description
Plot No.	We use the combination of the abbreviation of the administrative location and the sampling geographic location number to represent the number of the plot.
Plot No.- Species code	Combination of plot number and species number.
Family name	The full scientific name of the plant family.
Genus name	The full scientific name of the plant genus.
Species name	The full scientific name of the plant species.
Authors' name	Name of the person who named the species.
Classification System	The name of the plant classification system. In our case, it mainly is APG Ⅲ classification system.
Habit	The type of plant structure. In our case, it mainly includes Herb, Shrub and Subshrub.
Life cycle or leaf phenology type	Life cycle or leaf phenology of plants. In our case, it mainly includes Annual, Perennial and Deciduous.
Coverage (%)	It refers to the ratio of the projected area of a certain species in the Plot to the total area of land.
Leaflet area (cm^2^)	The leaf area of leaflets measured in centimeters squared (cm^2^).
Specific leaflet area (cm^2^/g)	The specific leaf area of the leaflets calculated by the ratio between leaf area (cm^2^) and leaf mass (g).
Leaflet dry mass (mg)	The mass of the dried leaflets measured in milligrams (mg). The data were converted to grams (g) for the calculation of specific leaflet area (SLA).
Leaflet nitrogen content per unit dry mass (mg/g)	The calculation method is to divide leaflet nitrogen (N) by the summed total dry mass of leaflets to obtain the nitrogen content (N) in the leaflets content per unit dry mass (LN; mg/g).
Petiole and rachis dry mass (mg)	The dry mass of the petiole and rachis. As the data are too small, in our case, "mg" is used as the unit of data record.
Petiole and rachis nitrogen content per unit dry mass (mg/g)	The calculation method is to divide petiole and rachis nitrogen (N) by the summed total dry mass of petiole and rachis to obtain the nitrogen content (N) in the leaflets content per unit dry mass (LN; mg/g).
